# Supplementary figures and images for: Enrichment of infection-associated bacteria in the low biomass brain bacteriota of Alzheimer’s disease patients
Source: PLoS One. 2024 Feb 9;19(2):e0296307. doi: 10.1371/journal.pone.0296307 (PMC10857729; doi:10.1371/journal.pone.0296307)

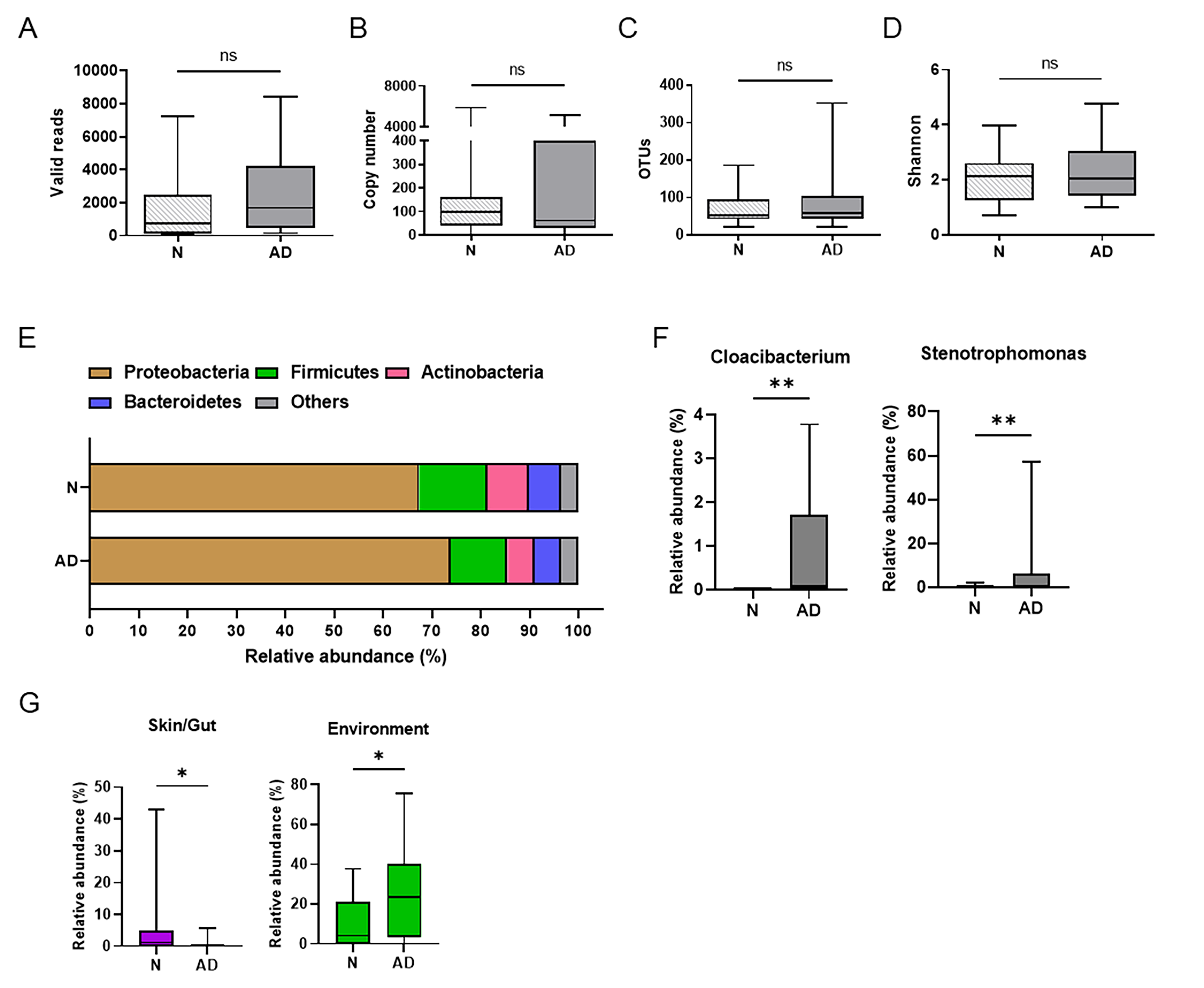

Supplement: S1 Fig — (A) Valid reads, (B) copy number, (C) OTUs, and (D) the Shannon index of N and AD are presented as box and whisker plots. (E) Compositions of phyla from brain samples are presented. (F) Relative abundance of the significantly enriched genus in N or AD is presented. (G) Relative abundance of the significantly enriched bacterial sources in N or AD is presented. The significance between N and AD was examined by the Mann–Whitney U test. (TIF) [file pone.0296307.s001.tif]

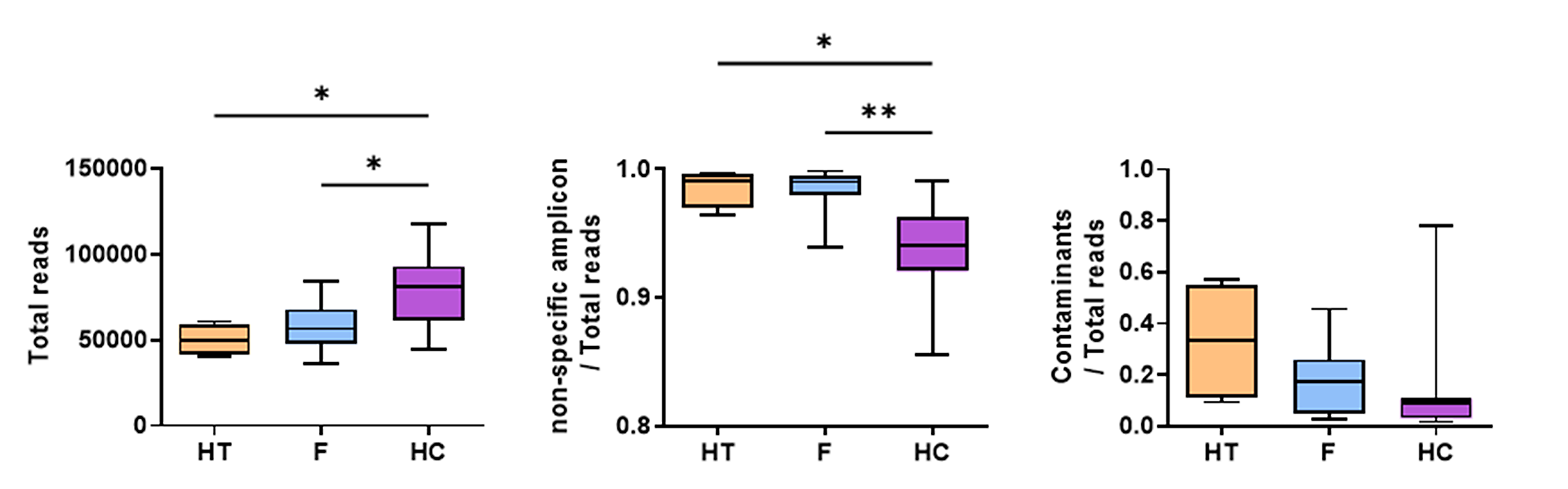

Supplement: S2 Fig — Total reads, non-specific amplicon/total reads, and contaminants/total reads in different brain areas are depicted. The significance among the HT, F, and HC was examined by the Kruskal–Wallis test. (TIF) [file pone.0296307.s002.tif]
