# Supplementary material for: Enrichment of infection-associated bacteria in the low biomass brain bacteriota of Alzheimer’s disease patients
Source: PLoS One. 2024 Feb 9;19(2):e0296307. doi: 10.1371/journal.pone.0296307 (PMC10857729; doi:10.1371/journal.pone.0296307)
Supplement: S1 Table — (DOCX) [file pone.0296307.s003.docx]

## Supplementary Table 1. Relative abundance of Top 50 genus in the brain areas of each subject type

|  | **N1-HT** | **N-F**  **Mean** | **N-HC**  **Mean** | **AD-HT**  **Mean** | **AD-F**  **Mean** | **AD-HC**  **Mean** |
| --- | --- | --- | --- | --- | --- | --- |
| ***Bradyrhizobium*** | 0.0 | 61.5 | 18.9 | 27.7 | 40.5 | 11.8 |
| ***Sphingomonas*** | 0.0 | 0.1 | 10.9 | 1.0 | 0.6 | 12.0 |
| ***Massilia*** | 10.2 | 0.1 | 0.1 | 3.8 | 1.3 | 18.1 |
| ***Staphylococcus*** | 10.2 | 4.9 | 2.2 | 15.5 | 1.5 | 0.3 |
| ***Pseudomonas*** | 0.0 | 0.7 | 2.7 | 1.2 | 10.8 | 1.7 |
| ***Caulobacter*** | 0.0 | 0.0 | 8.9 | 0.0 | 0.0 | 9.2 |
| ***Corynebacterium*** | 20.4 | 5.9 | 1.9 | 2.4 | 2.6 | 1.6 |
| ***Novosphingobium*** | 0.0 | 0.2 | 6.7 | 0.0 | 0.0 | 7.4 |
| ***Stenotrophomonas*** | 0.0 | 0.0 | 0.3 | 0.0 | 10.9 | 0.7 |
| ***Porphyromonas*** | 0.0 | 4.1 | 3.6 | 3.3 | 0.7 | 1.1 |
| ***Escherichia*** | 6.1 | 0.9 | 5.7 | 3.5 | 1.3 | 0.2 |
| ***Delftia*** | 0.0 | 0.0 | 4.4 | 1.1 | 0.3 | 5.9 |
| ***FM873692_g*** | 10.2 | 3.5 | 2.6 | 1.0 | 0.7 | 0.2 |
| ***Streptococcus*** | 0.0 | 1.3 | 1.0 | 6.8 | 1.7 | 0.8 |
| ***Acinetobacter*** | 6.1 | 1.6 | 1.6 | 0.2 | 2.9 | 0.4 |
| ***Bacteroides*** | 0.0 | 1.1 | 1.0 | 3.4 | 0.2 | 1.9 |
| ***Enhydrobacter*** | 0.0 | 0.8 | 1.8 | 2.4 | 0.5 | 1.2 |
| ***Enterobacteriaceae_g*** | 0.0 | 0.2 | 0.2 | 0.6 | 3.9 | 0.0 |
| ***Bacillus*** | 0.0 | 1.4 | 0.2 | 0.5 | 1.3 | 0.0 |
| ***Oceanobacillus*** | 0.0 | 0.0 | 0.0 | 0.0 | 3.0 | 0.0 |
| ***Faecalibacterium*** | 16.3 | 0.0 | 0.2 | 0.0 | 0.0 | 0.2 |
| ***Bartonella*** | 8.2 | 0.0 | 1.2 | 0.0 | 0.2 | 0.0 |
| ***Arthrobacter*** | 0.0 | 0.8 | 0.6 | 1.6 | 0.1 | 0.5 |
| ***Enterococcus*** | 0.0 | 0.0 | 0.0 | 0.0 | 2.0 | 0.1 |
| ***Thauera*** | 0.0 | 0.0 | 0.0 | 0.0 | 0.0 | 2.8 |
| ***Gemella*** | 0.0 | 0.4 | 0.4 | 2.1 | 0.0 | 0.4 |
| ***Prevotella*** | 0.0 | 0.3 | 0.9 | 0.0 | 0.3 | 0.6 |
| ***Lactobacillus*** | 0.0 | 0.0 | 1.4 | 0.0 | 0.4 | 0.0 |
| ***Bifidobacterium*** | 0.0 | 0.0 | 0.4 | 0.0 | 1.2 | 0.3 |
| ***Methylobacterium*** | 0.0 | 0.5 | 0.2 | 0.0 | 0.6 | 0.3 |
| ***Cetobacterium*** | 0.0 | 1.1 | 0.3 | 0.0 | 0.0 | 0.0 |
| ***Cloacibacterium*** | 0.0 | 0.0 | 0.0 | 0.0 | 0.9 | 0.8 |
| ***Sphingobium*** | 0.0 | 0.0 | 0.1 | 0.0 | 0.8 | 0.7 |
| ***Rothia*** | 0.0 | 0.9 | 0.4 | 0.0 | 0.0 | 0.1 |
| ***Phycicoccus*** | 6.1 | 0.0 | 0.2 | 0.1 | 0.0 | 0.2 |
| ***Savagea*** | 0.0 | 0.0 | 1.3 | 0.0 | 0.0 | 0.0 |
| ***Haemophilus*** | 0.0 | 0.3 | 0.0 | 2.0 | 0.0 | 0.1 |
| ***Agathobacter*** | 0.0 | 0.9 | 0.2 | 0.0 | 0.0 | 0.2 |
| ***Veillonella*** | 0.0 | 0.6 | 0.3 | 0.2 | 0.0 | 0.2 |
| ***Citrobacter*** | 6.1 | 0.1 | 0.1 | 0.0 | 0.0 | 0.1 |
| ***Acidovorax*** | 0.0 | 0.5 | 0.0 | 1.2 | 0.0 | 0.1 |
| ***Brevundimonas*** | 0.0 | 0.6 | 0.1 | 0.0 | 0.3 | 0.0 |
| ***Phreatobacter*** | 0.0 | 0.3 | 0.4 | 0.0 | 0.0 | 0.5 |
| ***Lactococcus*** | 0.0 | 0.0 | 0.9 | 0.0 | 0.0 | 0.0 |
| ***Actinomyces*** | 0.0 | 0.0 | 0.2 | 1.2 | 0.0 | 0.2 |
| ***Klebsiella*** | 0.0 | 0.0 | 0.0 | 0.2 | 0.7 | 0.1 |
| ***FJ893810_g*** | 0.0 | 0.0 | 0.0 | 0.0 | 0.8 | 0.1 |
| ***Diaphorobacter*** | 0.0 | 0.1 | 0.0 | 0.0 | 0.7 | 0.0 |
| ***Blautia*** | 0.0 | 0.0 | 0.5 | 0.1 | 0.0 | 0.2 |
| ***AB511016_g*** | 0.0 | 0.1 | 0.2 | 0.5 | 0.0 | 0.0 |
| **Others** | 0.0 | 4.3 | 14.9 | 16.3 | 6.1 | 16.9 |
