# Supplementary material for: Enrichment of infection-associated bacteria in the low biomass brain bacteriota of Alzheimer’s disease patients
Source: PLoS One. 2024 Feb 9;19(2):e0296307. doi: 10.1371/journal.pone.0296307 (PMC10857729; doi:10.1371/journal.pone.0296307)
Supplement: S2 Table — (DOCX) [file pone.0296307.s004.docx]

**Supplementary Table 2.** **Classification of species sources**

| **Oral** | **Oral/Gut** | | **Gut** | |
| --- | --- | --- | --- | --- |
| *Haemophilus parainfluenzae* | *Acinetobacter lwoffii* | *Streptococcus parasanguinis* | *Acidovorax delafieldii* | *Eggerthella lenta* |
| *Peptostreptococcus stomatis* | *Actinomyces naeslundii* | *Streptococcus salivarius* | *Acinetobacter guillouiae* | *Enterococcus cecorum* |
| *Porphyromonas pasteri* | *Actinomyces oris* | *Veillonella atypica* | *Aeribacillus pallidus* | *Eubacterium eligens* |
| *Prevotella melaninogenica* | *Bacteroides fragilis* | *Veillonella ratti* | *Agathobacter rectalis* | *Eubacterium hallii* |
| *Prevotella oris* | *Bacteroides plebeius* |  | *Agathobacter rectalis* | *Exiguobacterium aurantiacum* |
| *Ralstonia pickettii* | *Bacteroides stercoris* |  | *Akkermansia muciniphila* | *Holdemanella biformis* |
| *Streptococcus sanguinis* | *Bacteroides vulgatus* |  | *Akkermansia muciniphila* | *Lysinibacillus massiliensis* |
|  | *Bifidobacterium animalis* |  | *Alistipes putredinis* | *Massilia aurea* |
|  | *Bifidobacterium bifidum* |  | *Anaerostipes hadrus* | *Methylobacterium mesophilicum* |
|  | *Bifidobacterium catenulatum* |  | *Aquabacterium commune* | *Microbacterium phyllosphaerae* |
|  | *Bifidobacterium longum* |  | *Bacillus coagulans* | *Moraxella osloensis* |
|  | *Blautia glucerasea* |  | *Bacillus hisashii* | *Nakamurella intestinalis* |
|  | *Blautia wexlerae* |  | *Bacillus idriensis* | *Odoribacter splanchnicus* |
|  | *Corynebacterium riegelii* |  | *Bacillus infantis* | *Paenibacillus barengoltzii* |
|  | *Cryptobacterium curtum* |  | *Bacillus megaterium* | *Paenibacillus graminis* |
|  | *Dialister succinatiphilus* |  | *Bacillus mycoides* | *Parabacteroides goldsteinii* |
|  | *Fusobacterium nucleatum* |  | *Bacillus nealsonii* | *Parabacteroides merdae* |
|  | *Gemella haemolysans* |  | *Bacteroides acidifaciens* | *Paracoccus marinus* |
|  | *Lactobacillus fermentum* |  | *Bacteroides caccae* | *Proteus mirabilis* |
|  | *Lactobacillus gasseri* |  | *Bacteroides coprocola* | *Providencia stuartii* |
|  | *Lactobacillus johnsonii* |  | *Bacteroides dorei* | *Pseudomonas aeruginosa* |
|  | *Lactobacillus oris* |  | *Bacteroides faecichinchillae* | *Pseudomonas alcaligenes* |
|  | *Lactobacillus reuteri* |  | *Bacteroides intestinalis* | *Rhodococcus erythropolis* |
|  | *Lactobacillus ruminis* |  | *Bacteroides stercorirosoris* | *Ruminococcus faecis* |
|  | *Lactococcus lactis* |  | *Bacteroides stercoris* | *Ruminococcus torques* |
|  | *Neisseria mucosa* |  | *Bacteroides thetaiotaomicron* | *Savagea faecisuis* |
|  | *Neisseria subflava* |  | *Bauldia consociata* | *Shigella flexneri* |
|  | *Porphyromonas gingivalis* |  | *Bifidobacterium thermophilum* |  |
|  | *Prevotella copri* |  | *Blautia hansenii* |  |
|  | *Prevotella oris* |  | *Bradyrhizobium japonicum* |  |
|  | *Prevotellamassilia timonensis* |  | *Brevundimonas vesicularis* |  |
|  | *Rothia aeria* |  | *Butyricicoccus faecihominis* |  |
|  | *Ruminococcus bromii* |  | *Butyricimonas paravirosa* |  |
|  | *Ruminococcus gnavus* |  | *Clostridium bolteae* |  |
|  | *Streptococcus cristatus* |  | *Clostridium bolteae* |  |
|  | *Streptococcus cristatus* |  | *Clostridium perfringens* |  |
|  | *Streptococcus gordonii* |  | *Corynebacterium coyleae* |  |
|  | *Streptococcus mitis* |  | *Dorea formicigenerans* |  |

| **Environment-soil, water, air pollution** | | | |
| --- | --- | --- | --- |
| *Acidovorax soli* | *Ichthyenterobacterium magnum* | *Nocardioides furvisabuli* | *Shewanella profunda* |
| *Acinetobacter kookii* | *Intrasporangium mesophilum* | *Nocardioides kribbensis* | *Skermanella rosea* |
| *Albibacter methylovorans* | *Janibacter limosus* | *Nocardioides luteus* | *Sphingobium herbicidovorans* |
| *Alteromonas stellipolaris* | *Kineosporia rhamnosa* | *Nocardioides ungokensis* | *Sphingobium xenophagum* |
| *Arenimonas subflava* | *Knoellia aerolata* | *Noviherbaspirillum psychrotolerans* | *Sphingomonas adhaesiva* |
| *Arthrobacter bambusae* | *Kribbella antibiotica* | *Noviherbaspirillum suwonense* | *Sphingomonas daechungensis* |
| *Arthrobacter methylotrophus* | *Labrys soli* | *Novosphingobium ginsenosidimutans* | *Sphingomonas humi* |
| *Arthrobacter ramosus* | *Labrys wisconsinensis* | *Novosphingobium lindaniclasticum* | *Sphingomonas jaspsi* |
| *Azoarcus evansii* | *Lysobacter dokdonensis* | *Novosphingobium sediminicola* | *Sphingomonas kaistensis* |
| *Azoarcus tolulyticus* | *Lysobacter ginsengisoli* | *Ornithinimicrobium humiphilum* | *Sphingomonas limnosediminicola* |
| *Azohydromonas riparia* | *Lysobacter niabensis* | *Paenarthrobacter nicotinovorans* | *Sphingomonas sediminicola* |
| *Azohydromonas ureilytica* | *Lysobacter rhizophilus* | *Panacagrimonas perspica* | *Spirosoma rigui* |
| *Azorhizobium caulinodans* | *Lysobacter tyrosinelyticus* | *Paracoccus aminovorans* | *Stenotrophobacter terrae* |
| *Bacillus campisalis* | *Marmoricola aquaticus* | *Pedobacter ginsenosidimutans* | *Steroidobacter agariperforans* |
| *Blastococcus colisei* | *Marmoricola solisilvae* | *Pelomonas aquatica* | *Tardiphaga robiniae* |
| *Brevundimonas mediterranea* | *Marmoricola solisilvae* | *Phenylobacterium aquaticum* | *Thauera aminoaromatica* |
| *Brevundimonas staleyi* | *Marmoricola terrae* | *Phyllobacterium myrsinacearum* | *Thermomonas carbonis* |
| *Calidifontibacter indicus* | *Massilia agilis* | *Piscinibacter aquaticus* | *Treponema caldarium* |
| *Calidifontibacter indicus* | *Massilia arvi* | *Pleomorphomonas oryzae* | *Trichococcus flocculiformis* |
| *Caulobacter fusiformis* | *Massilia brevitalea* | *Porphyrobacter neustonensis* | *Variovorax defluvii* |
| *Caulobacter vibrioides* | *Massilia haematophila* | *Promicromonospora callitridis* | *Vicinamibacter silvestris* |
| *Cellulomonas uda* | *Massilia jejuensis* | *Pseudomonas azotoformans* | *Zoogloea ramigera* |
| *Chthoniobacter flavus* | *Massilia namucuonensis* | *Pseudomonas lini* |  |
| *Citrobacter portucalensis* | *Massilia norwichensis* | *Pseudomonas vancouverensis* |  |
| *Clostridium guangxiense* | *Massilia putida* | *Qipengyuania sediminis* |  |
| *Comamonas granuli* | *Massilia suwonensis* | *Ramlibacter solisilvae* |  |
| *Cryobacterium arcticum* | *Mesorhizobium huakuii* | *Ramlibacter tataouinensis* |  |
| *Cupriavidus necator* | *Mesorhizobium plurifarium* | *Ramlibacter_ginsenosidimutans* |  |
| *Dechloromonas denitrificans* | *Methylobacterium aquaticum* | *Rheinheimera aquatica* |  |
| *Deinococcus taklimakanensis* | *Methylobacterium extorquens* | *Rhizobium mongolense* |  |
| *Devosia insulae* | *Microvirga makkahensis* | *Rhizobium pusense* |  |
| *Devosia riboflavina* | *Moheibacter sediminis* | *Rhizobium zeae* |  |
| *Diaphorobacter nitroreducens* | *Mycobacterium sphagni* | *Rhodococcus fascians* |  |
| *Dokdonella kunshanensis* | *Nakamurella flavida* | *Rickettsiella popilliae* |  |
| *Flavobacterium amniphilum* | *Nakamurella multipartita* | *Rubellimicrobium mesophilum* |  |
| *Flavobacterium aquidurense* | *Nocardioides alpinus* | *Rubellimicrobium roseum* |  |
| *Hydrogenophaga bisanensis* | *Nocardioides aromaticivorans* | *Rubrivivax gelatinosus* |  |
| *Hymenobacter aerophilus* | *Nocardioides cavernae* | *Shewanella dokdonensis* |  |

| **Skin/Gut** | **Skin** | **Human others-clinical samples** | **Food** | **Others-animals, shower hose** |
| --- | --- | --- | --- | --- |
| *Corynebacterium amycolatum* | *Acinetobacter proteolyticus* | *Acinetobacter colistiniresistens* | *Pseudoalteromonas nigrifaciens* | *Acinetobacter towneri* |
| *Corynebacterium simulans* | *Staphylococcus petrasii* | *Anaerococcus tetradius* |  | *Altererythrobacter marinus* |
| *Staphylococcus aureus* |  | *Chryseobacterium shandongense* |  | *Azospirillum humicireducens* |
| *Staphylococcus epidermidis* |  | *Corynebacterium jeikeium* |  | *Bacillus hisashii* |
| *Staphylococcus hominis* |  | *Corynebacterium mucifaciens* |  | *Chryseobacterium vrystaatense* |
|  |  | *Corynebacterium pilbarense* |  | *Fibrobacter succinogenes* |
|  |  | *Corynebacterium riegelii* |  | *Paracoccus acridae* |
|  |  | *Corynebacterium vitaeruminis* |  |  |
|  |  | *Helicobacter macacae* |  |  |
|  |  | *Helicobacter macacae* |  |  |
|  |  | *Helicobacter pullorum* |  |  |
|  |  | *Klebsiella granulomatis* |  |  |
|  |  | *Massilia consociata* |  |  |
|  |  | *Massilia norwichensis* |  |  |
|  |  | *Massilia timonae* |  |  |
|  |  | *Mycobacterium simiae* |  |  |
|  |  | *Nocardia goodfellowii* |  |  |
|  |  | *Nocardia nova* |  |  |
|  |  | *Oblitimonas alkaliphila* |  |  |
|  |  | *Pseudomonas hunanensis* |  |  |
|  |  | *Pseudomonas oryzihabitans* |  |  |
|  |  | *Pseudomonas stutzeri* |  |  |
|  |  | *Roseomonas gilardii* |  |  |
|  |  | *Sphingobium yanoikuyae* |  |  |
