# Supplementary material for: Enrichment of infection-associated bacteria in the low biomass brain bacteriota of Alzheimer’s disease patients
Source: PLoS One. 2024 Feb 9;19(2):e0296307. doi: 10.1371/journal.pone.0296307 (PMC10857729; doi:10.1371/journal.pone.0296307)
Supplement: Attachment — Submitted filename: Rebutal letter.docx [file pone.0296307.s005.docx]

We appreciate the reviewers for their time and constructive comments on our manuscript.

Reviewer #1: The authors conducted 16S rRNA gene sequencing of 30 postmortem brain tissues from four individuals with normal and four AD patients. As the authors acknowledged, no specific bacterial species associated with AD pathology were identified that the authors had expected at the beginning of the study. However, the study is still significant on the point that some of the characteristics of the bacteria existing in AD and healthy brains were revealed. The quality of the paper would be improved if the following points were additionally mentioned.

1. Please comment as a limitation in the Discussion that the power to detect statistical significance is insufficient due to.

→ It has been added in the Discussion section as following (lines 372-374): However, this needs replication in future studies because the small number of specimens used in the current study limited the power to detect statistical significance.

2. Note that the fact that no significant difference does not suggest that there was no difference. For example, in the second paragraph of Overall structures of brain bacteria, “suggesting no overall differences in the bacterial communities of either frontal cortex or hippocampus between the N and AD groups”. This description may be misleading to readers and should be removed.

→ Removed.

3. Regarding classification 710 bacterial species into 10 categories in the Fig 4, I am not familiar with such classification. Are these classifications generally accepted? If so, please cite previous literatures related this classification. On the other hand, if the classification is made by the authors themselves, please submit the classification list as a Supplementary Table for future researchers to refer to.

→ The list has been provided as Supplementary Table 2. In its process, we discovered that the most abundant species *Bradyrhizobium japonicum* has been misclassified as oral/gut from gut, resulting in changes in Figs 4A and B.

Reviewer #2: General Comment. This is an interesting and well written paper about possible differences between the microbiota of Alzheimer’s and normal post-mortem brains. The authors report not finding any clear differences between these types of subjects in the areas of the brain sampled. They also report that their findings differ from what other researchers have found. My comments and questions are primarily focused on whether the author's methodology could account for the different findings and suggesting additional data details that enable a deeper understanding of the results. Specific questions and comments follow.

Contamination

The authors suggest that contamination of other researcher’s results could be an issue and specifically state that others have not sequenced any blank controls. One of these researchers in reference 18 specifically addresses the issue of contamination which we quote below, indicating that even if there were blank controls that showed contamination that they would not have been able to explain the large observed differences between AD and normal subjects, specifically Cutibacterium acnes.

“However, the consistently high levels seen here in AD samples compared to normal brains and the apparent minimal contribution of post mortem interval along with the lack of significant contact with skin makes contamination an unlikely explanation for the P. acnes content of these data.”

1) Could the authors explain why they think other researchers lack of blank controls seriously affected their results in comparison to the authors' new results.

→ *C. acnes* was identified in the initial data set of our study but filtered out in the exact binomial test in comparison with the three negative controls. Furthermore, the author of ref 18 reported increased levels of C. acnes based on the elevated read counts, which do not accurately represent the true microbial biomass. We discussed this in lines 379-388 of Discussion section as following: *Cutibacterium* (formerly *Propionibacterium*) *acnes*, a species previously suggested as a potential contributor to neuroinflammation (18), was identified in the initial dataset, reaching up to 5.5% (0 to 42 reads per sample), but was subsequently excluded as a contaminant in the filter 3. Emery et al. (18) interpreted the elevated bacterial reads (both total and *C. acnes*) in AD brains as indicative of increased bacterial levels. However, as mentioned earlier, read counts do not accurately represent the true microbial biomass. The predominance of *C. acnes* in AD brains was not replicated in a subsequent study by the same group (19), though differences in the brain regions studied (temporal cortex vs. hippocampus and locus coeruleus) could account for this inconsistency.

Bacteria Identification.

1) In reference 18, Emery et al. identify C. acnes (formerly P. acnes) as a bacterium whose abundance is very different between AD and N subjects. Could the authors explain why they do not report observing C. acnes?

2) Were there differences in the author’s sequencing protocol that could account for this? Could Cutibacterium (Propionibacterium) have been misidentified as something else? Here is a reference that addresses this somewhat.

Jacquelyn S. Meisel, Geoffrey D. Hannigan, Amanda S. Tyldsley, Adam J. SanMiguel, Brendan P. Hodkinson, Qi Zheng, Elizabeth A. Grice, Skin Microbiome Surveys Are Strongly Influenced by Experimental Design, Journal of Investigative Dermatology, Volume 136, Issue 5, 2016, Pages 947-956, ISSN 0022-202X,

https://doi.org/10.1016/j.jid.2016.01.016.,

link to useful supplemental table from this reference: https://ars.els-cdn.com/content/image/1-s2.0-S0022202X16003699-mmc1.pdf

I suggest that the authors discuss the above issues in a paragraph.

→ Thank you for providing a useful reference. According to the reference paper, *Propionibacterium* is underrepresented in V4 datasets compared to V1-V3 datasets. Because Emory et al. (ref 18) used primers targeting V3, and we used primers targeting V3-V4, the discrepancy is not likely due to a difference in the sequencing protocol. As described above, we discussed i) contamination issue, ii) read counts as insufficient indicator of biomass, and iii) variation in brain regions studied.

3) I understand that community statistical tests did not detect any significant differences between the various groupings. On the other hand, I did not see any lists of the main bacteria (genus level abundances) detected (except for a few examples) that would enable the reader to have a sense what was underlying these statistical computations. I suggest that the major bacteria detected be tabulated for the subjects/samples.

→ We included a heatmap for top 20 genera in each sample (Fig 3B) and the mean relative abundance of for top 50 genera in the brain areas of each subject type (Supplementary Table 1). The new result was described in the Result section (lines 275-277) and the most abundant genus *Bradyrhizobium* was discussed in the Discussion section (lines 395-398) as following:

[Result] At the genus level, the top 20 genera accounted for 40% to 96% of the total bacteriota, and the top 50 genera accounted for 83% to 100% of the total bacteriota, with *Bradyrhizobium*, a member of Proteobacteria, being the most abundant in many samples (Fig 3B and Table S2).

[Discussion] *Bradyrhizobium japonicum*, the sole species identified within the *Bradyrhizobium* genus, is known for its symbiotic relationship with legume roots but has also been identified in the human colon (28). Significantly, its association with prostate cancer, lung cancer, and colorectal cancer has been documented (28-30).

Bacterial Sources.

1) It would be useful to see tables of major bacteria (genus level abundances) that compare the brain areas of each subject type and the source locations for comparison purposes.

→ In Supplementary Table 2, the species classified into each bacterial source is listed.
